# Lost in translation: advancing intervention adaptation for populations with non-dominant language preference in high diversity settings

**DOI:** 10.1186/s43058-025-00753-6

**Published:** 2025-05-28

**Authors:** Kirsten Austad, Erika G. Cordova-Ramos, Alicia Fernandez, Mari-Lynn Drainoni

**Affiliations:** 1 Department of Family Medicine, Boston Medical Center and Boston University Chobanian & Avedisian School of Medicine, Boston, MA 02118 USA; 2 Evans Center for Implementation and Improvement Sciences, Department of Medicine, Boston University Chobanian & Avedisian School of Medicine, Boston, MA USA; 3 Department of Pediatrics, Boston Medical Center and Boston University Chobanian & Avedisian School of Medicine, Boston, MA USA; 4 Department of Medicine, University of California San Francisco, San Francisco, CA USA; 5 Section of Infectious Diseases, Department of Medicine, Boston Medical Center and Boston University Chobanian & Avedisian School of Medicine, Chobanian & Avedisian, Boston, MA USA; 6 Department of Health Law, Policy and Management, Boston University School of Public Health, Boston, MA USA

**Keywords:** Intervention adaptation, Non-dominant language preference, Cultural adaptation, Limited English proficiency

## Abstract

As the population of individuals with non-dominant language preference (NDLP) continues to grow, the field of implementation science has yet to fully address the unique barriers that this population faces in accessing evidence-based interventions (EBIs). Traditional models of cultural adaptation have been designed primarily for single linguistic or ethnic groups, focusing on aligning interventions with specific cultural values, beliefs, and practices. While effective within narrowly defined populations, this approach is not scalable to high-diversity settings where multiple NDLP groups are served simultaneously. In this Commentary, we argue for a reconceptualization of how implementation science approaches language barriers, advocating for all implementation efforts to consider language as a core determinant of success. We highlight how two relatively recent tools developed within implementation science—the Core Function and Form Framework and causal pathway diagrams—can advance EBI adaptation for populations with NDLP. We propose a highly scalable approach that systematically assesses the linguistic, cultural, and social needs of each individual and uses these data to guide individualized tailoring of an intervention, building on the emerging model of “personalized adaptation.” We highlight the need to innovate methods to ensure an individualized approach to EBI adaptation is feasible, scalable, and led by communities, with input from end-users. By harnessing the wisdom of the fields of implementation science and cultural adaptation, interventions can be adapted to the linguistic, cultural, and social needs of populations with NDLP to bring us closer to health equity in a diverse world.

## Background

**Fig. 1:**
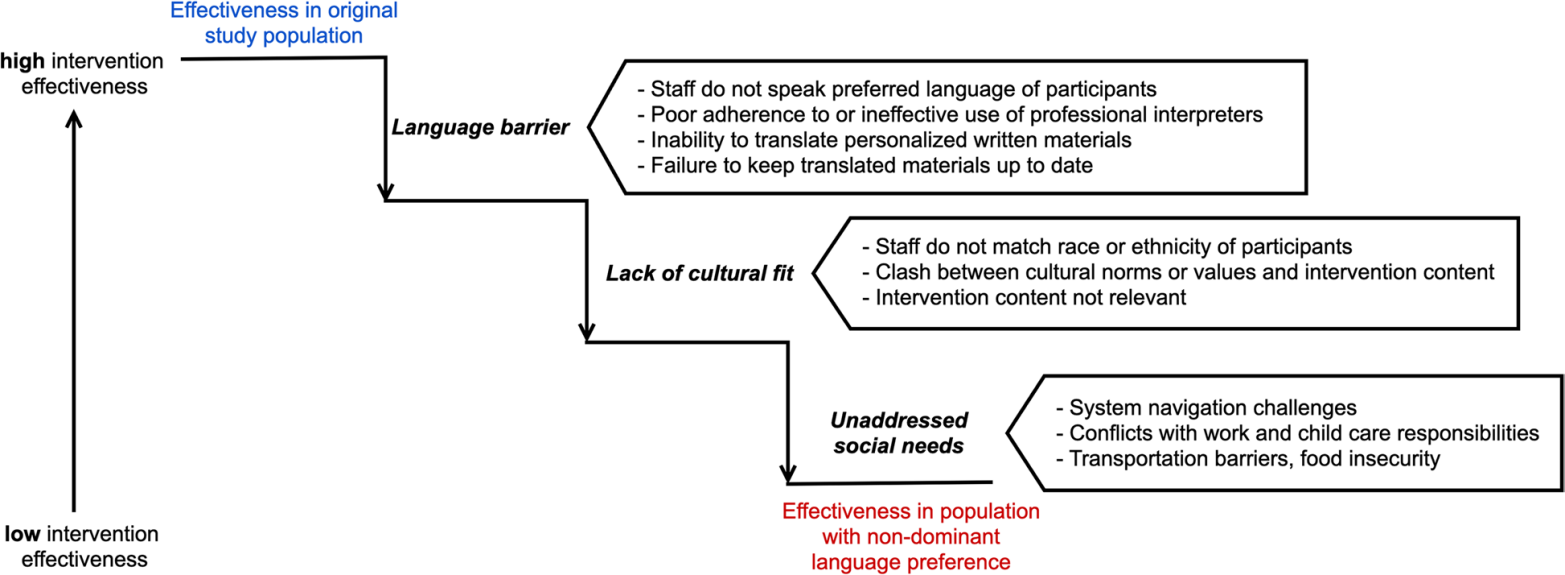
Drivers of lower effectiveness of evidence-based interventions (EBIs) when implemented in real world settings for populations with non-dominant language preference (NDLP)

Many studies focused on designing, testing, and implementing new interventions exclude individuals with a non-dominant language preference (NDLP) [1]. As a result, interventions are less effective for this population when rolled out in real-world settings where individuals speak many different languages (Fig. 1). The reason for these drop offs in effectiveness is not only related to language discordance—when the recipient and deliverer of an intervention do not share the same language—but also to two other interconnected barriers: lack of cultural fit and unaddressed social needs [2–4].

The role of language as a key determinant in implementation science (IS) has been largely overlooked. Most evidence-based interventions (EBIs) and implementation strategies rely heavily on communication, especially those that are patient-facing. Thus, individuals who are not proficient in the dominant language used to develop and test these interventions face substantial challenges to fully benefiting from them. The attenuated impact of unadapted EBIs applied to populations with NDLP may be mediated by decreased engagement and sustainment, lack of resonance of intervention content, or insufficient satisfaction or trust to catalyze behavior change.

Globally the total number of languages spoken is steadily decreasing, primarily due to a diminishing use of indigenous languages [5]. At the same time, rising immigration rates since the 1990s have led to a significant increase in language distribution within communities, meaning a single area now hosts speakers of numerous languages [6]. Even within a single linguistic group, considerable diversity exists according to country of origin, acculturation level, immigration experiences, and other dimensions. In the United States, where English is the predominant language, nearly one in four Americans now live in households where a language other than English is spoken, up from just one in ten in 1980 [7]. This growing linguistic diversity means that many agencies and systems must operate as “high diversity contexts,” requiring services to accommodate multiple NDLP subgroups simultaneously. While systematic data is lacking, such high diversity contexts are evident worldwide, from public schools in the United States to national referral hospitals in Guatemala.

As the NDLP population grows, so does the urgency to address barriers faced by this population in the real-world implementation of EBIs through proactive adaptation. While IS has included in its purview how to adapt EBIs to context, including characteristics of intervention recipients, overcoming implementation barriers related to language and culture have been largely deferred to the field of cultural adaptation. Cultural adaptation—defined as the “systematic modification of an evidence-based treatment (or intervention protocol) to consider language, culture, and context in a manner that aligns with the client’s cultural patterns, meanings, and values”—emerged from efforts to improve the fit of mental health EBIs for culturally minoritized populations including those with NDLP [8].

The complexity of high diversity contexts presents significant challenges when trying to adapt interventions for language and culture with the goals of enhancing participant engagement, content relevance, trust, and ultimately intervention effectiveness. Despite benefits demonstrated in research settings, culturally adapted interventions have not scaled to the extent needed to significantly reduce health inequities. By examining the barriers to scalability and realities of high-diversity contexts, we aim in this Commentary to reframe the approach to intervention adaptation for populations with NDLP within implementation science and offer recommendations for how to advance the science of adaptation for those with NDLP.

### Challenges to scaling culturally adapted interventions

**Fig. 2:**
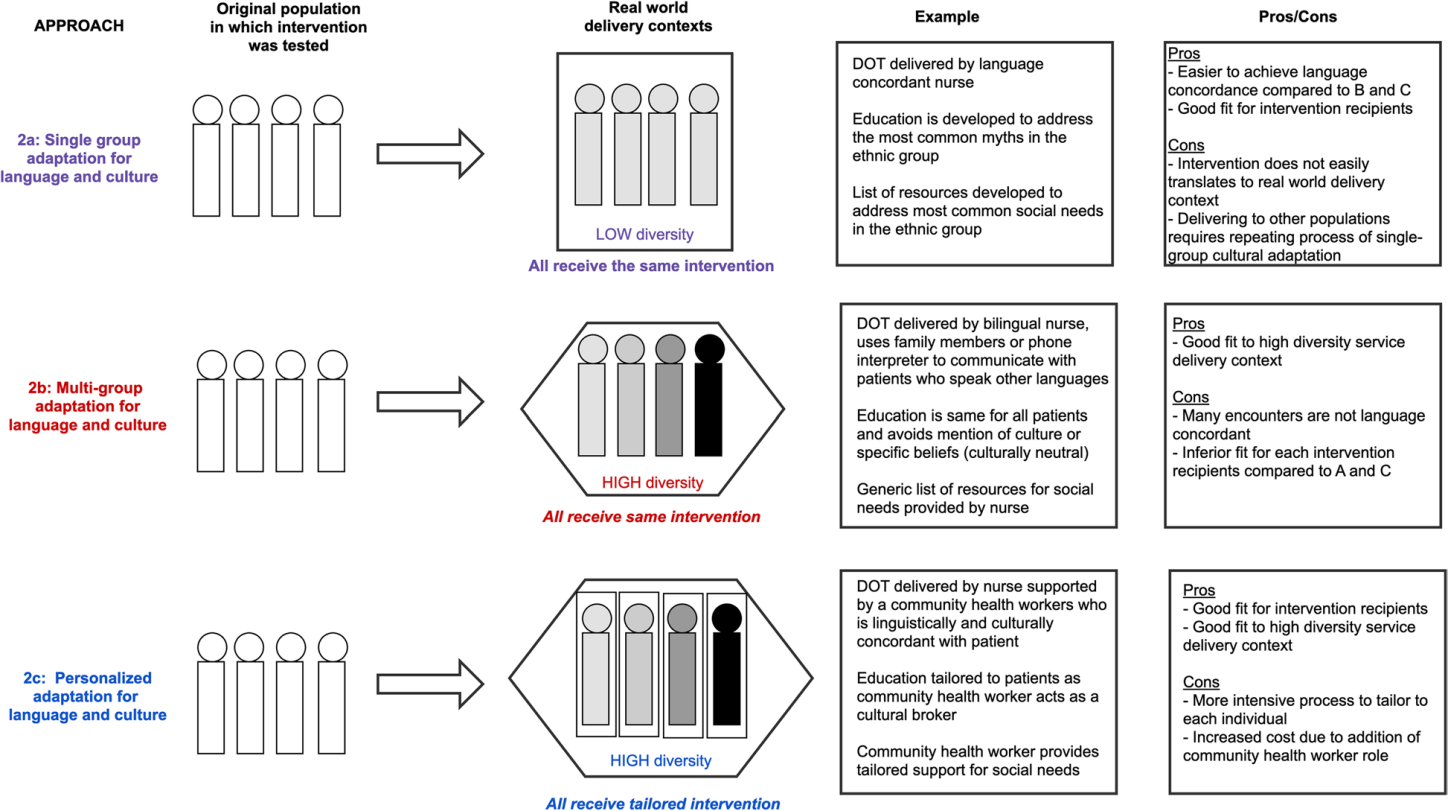
Analysis of the three approaches to cultural adaptation. Two current approaches to cultural adaptation (single group and multi-group) are compared with a version of personalized cultural adaptation proposed by the authors using visuals, examples, and analysis of pros and cons. The example case is a tuberculosis control program utilizing directly observed therapy (DOT)

Numerous systematic reviews have found that cultural adaptation moderately improves the effectiveness of EBIs for minoritized populations compared to the unadapted intervention [8–10]. Yet, these adapted interventions are rarely used outside of the trials in which they were designed. *One important reason why culturally adapted interventions have not been scaled is their misalignment with the real-world implementation contex*t [11]. The prevailing approach is single-group adaptation, meaning the intervention is tailored to fit the needs and cultural norms of one ethnic sub-group (Fig. 2a) [9]. The resulting adapted EBI can be delivered in a low diversity context serving only that subgroup, which is reasonable in settings where only one language or ethnic group far predominates. However, because of the mismatch between the tailored intervention and high diversity contexts which abound, it cannot be scaled to high diversity. In other words, traditional approach to cultural adaptation is not designed for broad-scale implementation. Imagine a hospital serving multiple linguistic groups that attempts to deliver a hospital discharge intervention to the four largest groups with NDLP. The inefficiencies of running four tailored interventions in parallel may make it logistically and financially infeasible, particularly in resource limited settings where linguistically diverse populations often receive care.

The likelihood of single-group adaptation to achieve the goal of reducing population level disparities is low for two reasons. First, carrying out cultural adaptation procedures for all demographic groups receiving services in a high diversity context is infeasible with current used methods that are complex, resource intensive, and owned by researchers. Iterative adaptation of EBIs for new minoritized populations produces “sideways progress” without necessarily advancing the science or reducing disparities on a larger scale [12]. Moreover, the dynamic nature of culture—especially when driven by waves of immigration—means that adapted interventions can quickly lose cultural fit.

### Intervention adaptation: beyond culture

The field of cultural adaptation has long recognized that structural factors can impede engagement with EBIs, such as those due to language barriers. A systematic reviews of psychosocial interventions found that intervention adaptation to create language-concordance are twice as effective as those that do not match provider and recipient language [13]. Despite this compelling evidence, the field has predominantly focused on aligning interventions with recipients’ cultural beliefs, often overlooking the logistic challenges of overcoming language barriers. In low diversity contexts, where the field of cultural adaptation has traditionally concentrated, addressing language barriers is relatively straightforward, as by hiring bilingual staff. However, in high-diversity contexts, where services must be delivered in multiple non-English languages simultaneously, solutions are far more complex. Without explicit focus on the logistical challenge of multilingual intervention delivery, the communication barrier is not explicitly addressed as part of intervention adaptation. The result is that language barriers are overcome with last-minute solutions which often have lower acceptability or are implementing with low fidelity, ultimately undermining EBI effectiveness.

A second structural barrier to EBI engagement and effectiveness is health-related social needs (HRSN). While HRSN are not exclusive to individuals with NDLP, they are more prevalent among linguistically minoritized groups and thus drive disparities at the population-level. In low-diversity settings, practitioners can become facile in addressing HRSN through knowledge of community resources. In high-diversity contexts, however, the variety of HRSN, resources potentially targeted to different groups, and different qualifications (e.g. citizenship) for resources strain practitioners. *If language and HRSN are not systematically addressed as part of an integrated cultural adaptation strategy for populations with NDLP, the impact of adaptation will be attenuated in high diversity contexts.*

### Potential solutions

Continuing the current approach to adapting interventions for language and culture is unlikely to achieve the reduction in health disparities needed to achieve equity for communities with NDLP. *What is needed is an approach that achieves fit to the recipient’s linguistic, cultural and health-related social needs as well as the service context.* We propose that this can be best accomplished by integrating the expertise of implementation science (IS)—particularly in addressing implementation barriers and designing for dissemination—with the foundational contributions of the field of cultural adaptation. To that end, we now highlight existing tools—and those that are still needed—to achieve a more scalable approach to adaptation for language and culture (Table 1).

**Table 1:** Recommendations for advancing intervention adaptation for populations with non-dominant language preference

***Utilize implementation science tools that promote replicability and generalizability***
Track intervention adaptations using the language, culture, and social needs categorization
Apply the Core Function and Form Framework to identify the core functions of an intervention for which specific forms may differ across high diversity contexts
Propose and test a theory of the mechanism by which culturally adapted implementation strategies (or interventions) achieve their intended impact using causal pathway diagrams (or corollary approaches)
***Develop the science to allow for personalized intervention adaptation***
Develop a rapid method for intervention adaptation for language and culture
Innovate methods for community-led “last mile” intervention adaptation for language and culture
Create and validate brief patient-reported measures of cultural fit

### Use of the core functions and form framework

Cultural adaptation has long recognized the fidelity-adaptation paradox—the tension between adapting interventions to improve cultural fit while maintaining the core components for effectiveness—that mirrors the debate in IS between generalizability with context [14]. Both disciplines acknowledge that adaptation is essential (i.e., strict intervention fidelity should not be the goal), but have struggled to provide clear guidance on how to ensure that effectiveness is preserved during adaptation.

The Core Functions and Forms Framework (CFFF) offers a promising path forward for the adaptation of complex interventions [15]. The CFFF differentiates between an intervention's core functions (the fundamental purposes or desired outcomes) and the forms (specific activities or practices) used to achieve those functions. The goal in intervention adaptation is to retain the core functions but allow the forms to be tailored to the target population and other aspects of context. A marked advantage of this approach over prior work in IS—such as the distinction between “core components” and “adaptable periphery”—is that whether an adapted intervention achieves the intended function can be measured. Examples of how to operationalize the CFFF in low and high diversity settings to facilitate adaptation for populations with NDLP are provided in Table 2.

**Table 2:** Analysis of adaptation for populations with non-dominant language preference of two different evidence-based interventions

	Example 1: Hospital Discharge		Example 2: Neonatal Intensive Care Unit (NICU)	
**Evidence-based intervention (EBI)**	Complex intervention to improve transitions of care from hospital to home (hospital discharge)		Intervention to promote breastfeeding in the NICU setting through peer education and support counselors	
**Intervention core FUNCTIONS**	1. Provide understandable self-care instructions 2. Educate on medication regimen, adherence, and potential side effects 3. Identify and answer post-discharge questions		1. Educate on benefits of breastfeeding in preterm infants 2. Provide emotional and logistical support to facilitate breastfeeding 3. Identify and address barriers to caregiver presence in NICU	
**Need for intervention adaptation**				
**Language**	- Language concordant educator (vs. interpreter) - Increased challenge of using teach back strategy to confirm understanding - Translation of personalized written instructions		- Language concordant educator (vs. interpreter) - Translation of standardized educational handouts	
**Culture**	- Preferences for involvement of family - Beliefs related to medications - Explanatory models for illness		- Align recommendations with cultural norms of breastfeeding - Address myths about breastfeeding specific to sub-groups	
**Social needs**	- Lack of health insurance or underinsurance - High out of pocket costs of medications - Follow up constraints due to missed work		- Frequent transportation to hospital to breastfeed or bring pumped milk - Access to quality breast pump and pumping supplies	
**Context**	***Low diversity context***	***High diversity context***	***Low diversity context***	***High diversity context***
**Intervention FORMS**	1. Delivered by a Spanish speaking nurse trained in teach back^a^ 2. Translation in Spanish done by nurse 3. Nurse links patients to resources based on her knowledge of Spanish speaking community	1. Spanish speaking nurse, also trained in interpreter use with teach back focus 2. Audio recorded by interpreter as unable to arrange for translation in all languages 3. Systematic social determinants of health screening and resource binder	1. One version of educational materials that present common misbeliefs 2. Spanish speaking counselor 3. Parking voucher	1. Different educational materials target misbeliefs in each sub-group 2. Core Spanish speaking counselor plus per diem on call counselors for less common linguistic groups 3. Vouchers for parking, ride-sharing app, or public transportation

The chart presents two example EBIs, both complex interventions, and how they require adaptation due to barriers stemming from language, culture, and social needs common among patients with non-dominant language preference. Each intervention is defined as having core functions which are achieved through specific intervention forms, which will be accomplished differently in low and high diversity contexts. The low diversity context is primarily Spanish speaking while the high diversity context has numerous linguistic sub-groups that receive care in parallel

^a^Approved bilingual provider

### Causal pathway diagrams

To improve replicability and generalizability, experts in cultural adaptation have emphasized the need for a deeper understanding of the mechanisms through which cultural adaptation enhances effectiveness of interventions. However, no clear methodology has emerged. The recent development of causal pathway diagrams (CPDs**)** in IS offers a promising approach [16]. CPDs provide a structured method for articulating the specific mechanisms by which an implementation strategy achieves its intended outcomes. Currently CPDs are only used to analyze implementation strategies, but a similar approach to detail the causal mechanisms of core functions may also advance the field.

**Fig. 3:**
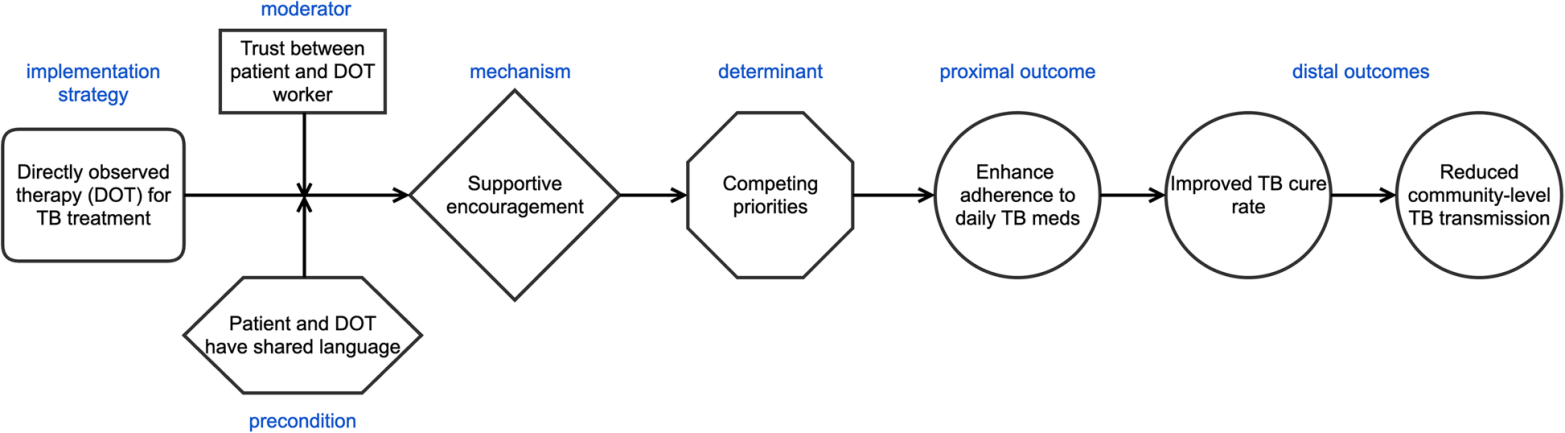
An example causal pathway diagram that depicts their utility in identify moderators and preconditions that are salient in high diversity contexts. As with Fig. 2, the example presented is for directly observed therapy (DOT) as a strategy to increase adherence to tuberculosis (TB) treatment

Integrating CPDs into efforts to adapt interventions for language and culture has two key benefits. First, identifying the mechanisms underlying implementation strategies allows for more replicable adaptation, and therefore retained intervention effectiveness. CPDs identify both proximal and distal implementation outcomes which can (and should) be measured as a signal that the adapted implementation strategy is working as intended. Second, CPDs help pinpoint contextual factors-- “preconditions” and "moderators"-- that must be addressed for implementation strategies to succeed [16]. To illustrate these benefits, consider the example of directly observed therapy (DOT) for treatment of tuberculosis (Fig. 3). By identifying shared language between the patient and health worker performing DOT as a precondition, implementers can anticipate challenges that will negatively impact deployment in linguistically diverse populations.

### Moving beyond single-group adaptation in high diversity contexts

Shifting to a new approach to adaptation for language, culture, and social needs compatible with high diversity contexts could better align interventions with real world situations, thereby improving scalability, sustainability, and ultimately impact on health disparities for populations with NDLP. One existing alternative is multi-group adaptation (Fig. 2b), which delivers EBIs to heterogenous linguistic and cultural group simultaneously primarily by removing elements that might clash with specific cultural values. The result is often considered a "culturally neutral” intervention. While it produces an intervention aligned with the service delivery context, multi-group adaptations have a lower impact compared to single-group adaptations [9]. Furthermore, multi-group adaptation does not address language barriers and social needs systematically.

A better model would be one that, like multi-group adaptation, can be deployed in a high diversity context while also achieving good fit for culture and linguistic and social needs. We propose an approach in which a pre-intervention assessment identifies the norms and needs within the domains of culture, language, and HRSN of each individual, and uses this information to deliver a personalized intervention (Fig. 2c). The degree of modifications made between participants would depend on the specific intervention and range from small, e.g. written materials in different languages, or large, e.g. adding a new component to an intervention to connect participants to resources for HRSNs (Table 2) [17].

An emerging model of “personalized adaptation” aligns with our vision. Given disparities in engagement and outcomes among minoritized groups, Yeh and colleagues used personalized adaptation to tailor Parent–Child Interaction Therapy (PCIT) for culturally diverse families [18]. Caregivers underwent a baseline assessment to understand their treatment expectations, etiological explanations, parenting styles, and family support. Therapists then personalized PCIT, using this data to better align the intervention with their beliefs. For example, parents identified as having an authoratative parenting style were provided with additional education during PCIT sessions on drawbacks to this style. By improving the alignment between the parents' beliefs and the therapeutic approach​, personalized adaptation PCIT (compared to unadapted PCIT) improved parental motivation and children’s behavioral outcomes.

We propose developing an approach of personalized adaptation that incorporates tailored elements for linguistic and social needs and testing it across a broad range of EBIs. However, for personalized adaptation to be scaleable, it must be feasible, which requires new methodologies and tools.

### Methods to make personalized adaptation feasible

Resource intensive qualitative methods are often used to inform the adaptation of interventions for culture [19]. Regardless of the approach to cultural adaptation, carrying it out faster would improve the reach of adapted interventions. We can build on recent advances in “rapid” methods—including rapid qualitative analysis and rapid ethnographic assessment—which have been shown to produce equally rigorous conclusions with less time and resources [20, 21]. Integrating these rapid methods into cultural adaptation processes, along with the development of new ones, could provide more feasible ways to tailor interventions.

While many have advocated for a more community-driven process of intervention adaptation, it is challenging to operationalize that due to the specialized, resource intensive methods used. To improve scalability, new approaches to adapt interventions for populations with NDLP should empower end users—such as community organizations and ethnocentric health centers—to lead adaptation and evaluation efforts. Such an approach would align well with personalized adaptation, as researchers could define the base intervention and tailor it in collaboration with local leaders, who then make final adjustments (the “last mile” of the adaptation process) and deliver it.

Lastly, to ensure intervention recipients have a voice in the adaptation, there is a need for brief, validated patient-reported outcome measures for perceived cultural fit. Potential constructs addressed could include message resonance, familiarity of word choice in written materials, and trust. Such measures can also serve as early signals of whether an adapted intervention is achieving its desired goal early in implementation to allow for course correction.

### Challenges ahead

Personalized adaptation offers promise for improving intervention fit in high diversity contexts but requires careful evaluation as to whether effectiveness gains outweigh the complexity it introduces. This balance likely varies by both context and the EBI itself. Some interventions may require only minimal modifications for language and culture, while others deeply embedded in culturally and linguistically “sensitive” topics may require significant changes and attain greater benefit from personalized adaptation. Drawing parallels from health-related social needs [22], which has sought to identify specific high-impact conditions for which addressing social determinants is most critical, we should determine when personalized adaptation yields the greatest returns. Similarly, the tools we recommend to improve generalizability, such as the CFFF, may prove more time-intensive than anticipated, particularly when seeking consensus among diverse stakeholders. These feasibility challenges underscore the critical importance of rigorous evaluation to ensure efforts to reduce disparities among populations with NDLP are successful.

Conducting randomized trials of all adapted interventions is infeasible and would divert funds from other health equity efforts; we join others in advocating for better utilization of real-world data to this end. Learning health systems—healthcare systems that continuously collect and analyze data from clinical practice to generate evidence, which is then rapidly integrated back into care processes to improve patient outcomes [23]—could prove useful in conducting real world evaluations of interventions adapted for populations with NDLP, allowing for ongoing refinement and optimization of strategies to reduce health disparities. When randomized trials are needed, type two or three hybrid effectiveness-implementation designs should be utilized.

## Conclusion

To address the growing cultural diversity and social needs of populations with NDLP, it is crucial to reimagine current approaches to cultural adaptation of EBIs. While cultural adaptation has shown promise in improving engagement and outcomes by tailoring interventions to ethnic subgroups, in high diversity contexts when interventions must serve participants from varied backgrounds and languages simultaneously, this prevailing approach is impractical. The resulting mismatch between the intervention and the real-world service delivery context precludes EBI integration into usual care.

Moving forward, personalized adaptation offers a promising model to tailor interventions to the needs of those with NDLP. By understanding the unique linguistic, cultural, and social needs of each individual, better intervention fit can be achieved. By combining the strengths of the fields of cultural adaptation and IS, we can develop interventions that better meet the needs of populations with NDLP and address the systemic barriers that give rise to health inequities.

## Data Availability

Not applicable.

## Abbreviations

CPD: Causal pathway diagram
DOT: Directly observed therapy
EBI: Evidence-based intervention
HRSN: Health-related social need
NDLP: Non-dominant language preference
PCIT: Parent–Child Interaction Therapy
TB: Tuberculosis
US: United States
